# A Selective Screening Strategy Performed in Pre-School Children and Siblings to Detect Familial Hypercholesterolemia

**DOI:** 10.3390/children9050590

**Published:** 2022-04-21

**Authors:** Alexandra Thajer, Margot Baumgartner, Anselm Jorda, Ulrike Hallwirth, Julia Lischka, Susanne Greber-Platzer

**Affiliations:** 1 Department of Pediatrics and Adolescent Medicine, Division of Pediatric Pulmonology, Allergology and Endocrinology, Medical University of Vienna, 1090 Vienna, Austria; alexandra.thajer@meduniwien.ac.at (A.T.); margot.baumgartner@meduniwien.ac.at (M.B.); anselm.jorda@meduniwien.ac.at (A.J.); julia.lischka@meduniwien.ac.at (J.L.); 2 Municipal Authority of the City Vienna, Municipal Department 15, Health Service of the City of Vienna, 1030 Vienna, Austria; ulrike.hallwirth@wien.gv.at

**Keywords:** familial hypercholesterolemia, screening, LDL-cholesterol, pre-school children, prevention, premature cardiovascular event

## Abstract

(1) Background: Familial hypercholesterolemia (FH), a most common genetic disorder, is underdiagnosed and untreated, especially in children. Individuals with heterozygous familial hypercholesterolemia mostly present without clinical symptoms and are not informed about their high risk for myocardial infarction. Early diagnosis and treatment can prevent premature atherosclerosis and cardiovascular events in patients with FH. The aim was to evaluate the detection rate of pre-school children with FH at school doctor visits in Vienna and, moreover, to examine the frequency of FH identified in the children’s siblings by this type of screening. (2) Methods: The selective FH- screening was implemented at the school enrolment examinations in the public primary schools of Vienna. The study period included the school years starting in 2017 to 2020. FH was suspected if a questionnaire on hypercholesterolemia, or cardiovascular events in the family history or on the presence of xanthomas or xanthelasma, was positive. Subsequently, lipid testing was performed on pre-school children and their siblings and elevated lipid screening was defined as either positive by LDL-C ≥ 160 mg/dL and/or non-HDL-C ≥ 190 mg/dL or as borderline by LDL-C ≥ 130 mg/dL and/or non-HDL-C ≥ 160 mg/dL. (3) Results: 66,108 pre-school children participated in the school enrolment examination in 868 public elementary schools in Vienna. In 512 (4%) children, the questionnaire caused suspicion of FH. 344 families agreed their participation in the study. Out of 344 (52% male) tested pre-school children, 20 individuals (40% male) had elevated blood lipid levels with a mean LDL-C of 155 ± 29 mg/dL and a non-HDL-C of 180 ± 24 mg/dL. Out of 291 (44% male) tested siblings, 17 individuals (41% male) showed elevated lipids with a mean LDL-C of 144 ± 19 mg/dL, and a non-HDL-C of 174 ± 19 mg/dL. (4) Conclusions: Screening is the key for early diagnosis and treatment of FH. We have implemented a pre-school screening strategy in cooperation with school physicians. We could identify 20 pre-school children and 17 siblings with an elevated lipid screening test. Full implementation of FH-screening in the pre-school examination visits in Vienna would help to detect high-risk children.

## 1. Introduction

Familial hypercholesterolemia (FH) is an autosomal dominant recessive inherited disease and every minute, a heterozygous child is born worldwide [1,2]. The prevalence of heterozygous familial hypercholesterolemia (heFH) is estimated to be between 1 per 200 and 1 per 500 in Europe [2,3]. This most common genetic disorder is underdiagnosed and untreated, especially in children [2]. Therefore, it is fundamentally important to increase the awareness of familial hypercholesterolemia in the general population and in medical professionals. A recent review showed that an improvement is noticed in the development of existing models of care for FH and that a collaboration with the WHO is the next step to successfully close the knowledge gap [4]. The main characteristics of this hereditary disease are lifelong increased low-density lipoprotein cholesterol (LDL-C) levels from birth onwards, possible positive family history of premature cardiovascular complications, and possible presence of tendon xanthomas, xanthelasmata, and arcus lipoides (2). However, a main issue is that especially individuals with heFH, do not suffer from any symptoms early in life, which makes detection of this monogenetic disorder more difficult. The diagnosis can be confirmed clinically (by elevated LDL-C levels and family history) or genetically. In general, FH is an autosomal dominant disease caused by mutations in the genes coding for LDL-receptor (*LDLR*), apolipoprotein B (*APOB*) or proprotein convertase subtilisin kexin type 9 (*PCSK9*), or autosomal recessive related to the low-density lipoprotein receptor adaptor protein 1 (*LDLRAP1*) [2,5,6]. In 5–30% of all phenotypic FH cases, no pathogenic mutation in these genes can be found. Therefore, polygenetic causes or unknown genes are suspected [7]. The diagnosis of pediatric heFH patients is based on family history concerning hypercholesterolemia, LDL-C cut-off levels, and/or premature cardiovascular disease (CVD) [8]. Untreated patients have a 100-fold increased risk to develop coronary heart disease (CHD) in comparison to the unaffected population [9]. Moreover, untreated children suffering from heterozygous familial hypercholesterolemia have an increased premature CHD risk after the second decade of life [10]. The purpose is the prevention of atherosclerosis and cardiovascular diseases in early life. Therefore, early detection, diagnosis, and adequate treatment of this public health burden are essential.

Screening is the key to fight against the harmful effects of familial hypercholesterolemia. Different screening options are possible, from universal to cascade to selective screening strategies, as well as combinations, e.g., universal and reverse cascade screening. A role model of cascade screening has been successfully implemented in the Netherlands for 20 years [1]. In Slovenia, a universal screening has been initiated for children at the age of five years, performed by primary pediatricians within their routine examination [11,12]. However, for each country, only a feasible screening method can be established. In Austria, we do not have the support of either the health ministry or primary pediatricians, nor a compulsory vaccination program in young children to reach this specific population. Detection as early as possible is essential for individuals affected by familial hypercholesterolemia to start lipid-lowering therapy and to decrease the risk for premature cardiovascular pathology.

In Vienna, pre-school children have a physical examination prior to school enrolment performed by school physicians in all public elementary schools. Thus, we have set up a selective FH-screening in pre-school children in cooperation with school physicians performed at pre-school examinations. In 2017, this novel screening tool for familial hypercholesterolemia in children has been started and the outcomes of the first-year results have been previously published [13]. This selective FH-screening strategy seems to be the right step to detect this common disease as public health issue in young aged children and has been therefore continued.

The aim of the study was to evaluate the detection rate of pre-school children with FH performed at the school doctor visits in Vienna for the last four years and, moreover, to examine the frequency of FH identified in the siblings by this type of screening.

## 2. Materials and Methods

### 2.1. Study Design and Recruitment of Study Population

All school physicians have received training and all study documents prior to the study initiation. This selective screening, also called FHkids, was performed at school enrolment examinations in all public primary schools in Vienna between January 2017 and May 2020. The first step was that school physicians administered a questionnaire on family history for the parents. The three key questions were about elevated lipids or lipid-lowering medication, familial premature cardiovascular complications in parents as well as in first- or second-degree relatives, and if parents ever had signs of xanthomas or xanthelasma. In addition, a picture of xanthomas was utilized for visualization and explanation of these skin lesions. Details of the school physician questionnaire have been previously published [13]. The school physician questionnaire was available in the languages German, Turkish, English, and Bosnian-Croatian-Serbian to reach as many families as possible. A questionnaire was defined as positive when one of the questions was answered with “yes” and/or one of the questions could not be answered or was unknown. 

The next step was that all families, including pre-school children and their siblings, with a positive questionnaire were invited to the Department of Pediatrics and Adolescent Medicine at the Medical University of Vienna. Then an individual questionnaire, also available in four languages, was completed with each parent. 

The mothers’ and fathers’ questionnaire included the following questions:

  (1) Do you have elevated blood fats (=total cholesterol, triglycerides, LDL-cholesterol)?

      O     Yes      O     No      O     Not measured

  (2) Do you take cholesterol-lowering medication?

    O    Yes    O    No
    If the answer to this question is yes, please specify medication (indicate medication and daily dosage): _________________________________________________

  (3) Do you have fatty skin growths/deposits (=xanthomas, xanthelasms) above all in the areas of the Achilles tendon/hands/knees or eyes?

      O     Yes        O     No

  (4) Do you suffer from heart diseases (=calcification/narrowing of the coronary vessels, heart attack), narrowing of large vessels (e.g., main artery, carotid artery) or cerebral circulatory disorders (=stroke)?

      O     Yes      O      No        If yes, indicate the disease/disorder: ____________

  (5) Do close relatives (first and second grade*) have:

         -elevated blood fats (=total cholesterol, triglycerides, LDL-cholesterol)

      O     Yes      O      No        If yes, indicate the relatives: __________________

         -Have close relatives (first and second grade*) suffered a heart attack before 

      the age of 50 (men) or 60 (women)?

      O     Yes      O      No        If yes, indicate the relatives: ___________________

      *First-grade relatives: children, parents, and siblings. Second-grade relatives: 

      grandparents, grandchildren, uncles, aunts, nephews, nieces, and half-sisters.

The parents’ questionnaire was completed prior to the children’s lipid screening test and therefore, the study team was able to clarify open questions with the parents immediately. This ensured that all questions from mothers and fathers were answered. After completing the questionnaires, the lipid screening test was carried out on the pre-school children and their siblings. Prior to the study participation, all families were informed and a written informed consent form, also available in four different languages, was obtained from all parents or legal representatives. This study was approved by the Institutional Review Board of the Medical University Vienna (EC Nr: 2019/2015).

### 2.2. Lipid Screening Test

The Alere Afinion^TM^ AS 100 Analyzer (Alere GmbH, Linz, Austria) was used for quantitative measurement of the lipid profile. Alere Afinion^TM^ Lipid Panel Test Cartridge (Alere GmbH, Linz, Austria) was utilized to measure total cholesterol (TC), high-density lipoprotein cholesterol (HDL-C), and triglycerides (TG). The analyzer calculated low-density lipoprotein cholesterol (LDL-C), non-HDL cholesterol (non-HDL-C), and cholesterol/high-density lipoprotein cholesterol ratio (C/HDL-C ratio). The measuring range of Alere Afinion^TM^ AS 100 Analyzer (Alere GmbH, Linz, Austria) is specified for total cholesterol between 100 to 500 mg/dL, HDL-C between 15 to 100 mg/dL and triglycerides between 45 to 650 mg/dL. All test kits were stored according to the manufacturer’s instructions in the refrigerator at +2 to +8 °C. The kits were required to reach room temperature, between +15 to +25 °C, prior to each finger prick test, and thus, test kits were removed from the refrigerator before testing. For each test, a sample volume of 15 µL capillary blood, directly from the finger, was needed. Results were provided in about eight minutes. An elevated lipid screening test was defined as either positive by LDL-C ≥ 160 mg/dL and/or non-HDL-C ≥ 190 mg/dL or as borderline by LDL-C ≥ 130 mg/dL and/or non-HDL-C ≥ 160 mg/dL. 

Lipid screening testing was performed at the Department of Pediatrics and Adolescent Medicine at the Medical University of Vienna. All pre-school children and siblings with a positive lipid screening test were invited to the outpatient clinic of obesity, lipometabolic disorder, and nutritional medicine at the Department of Pediatrics and Adolescent Medicine, Medical University of Vienna. This ensured that children with familial hypercholesterolemia received an appropriate treatment and that venous blood measurement in a specialized lipometabolic disorder outpatient clinic was performed.

For diagnosis, genetic testing was offered for all known disease-causing gene variants. The DNA was isolated from EDTA-anticoagulated blood and was sequenced using Amplicon Sequencing (SEQPRO Lipo; Progenika) on the MiSeq-system (Illumia). Genetic testing of the following genes associated with familial hypercholesterolemia were fully or partially sequenced as indicated: LDL-receptor (*LDLR*), proprotein convertase subtilisin/kexin type 9 (*PCSK9*), Apolipoprotein B (*APOB*) (exon 26 c.10438-10757 and exon 29 c.13009-13301 only), Apolipoprotein E (*APOE*) (c.225-521 only), and low-density lipoprotein receptor adaptor protein 1 (*LDLRAP1*).

### 2.3. Statistical Analysis

Data were expressed as mean ± standard deviation or as median with range. Categorical data were presented as absolute frequencies and proportions. The unpaired t-test was performed to determine gender differences. The paired t-test was used for comparison of capillary blood (lipid screening test) and venous blood (lipometabolic disorder specialized outpatient clinic) results. A *p*-value < 0.05 was considered as an indicator of statistical significance. Statistical analyses were performed using the software Statistical Package for Social Science (SPSS 26.0, IBM Corporation, New York, NY, USA).

## 3. Results

### 3.1. FH-Screening Strategy

From 2017 to 2020, 260 school physicians have performed school enrolment examinations on 66,108 pre-school children in 868 public elementary schools in Vienna.

**Figure 1:**
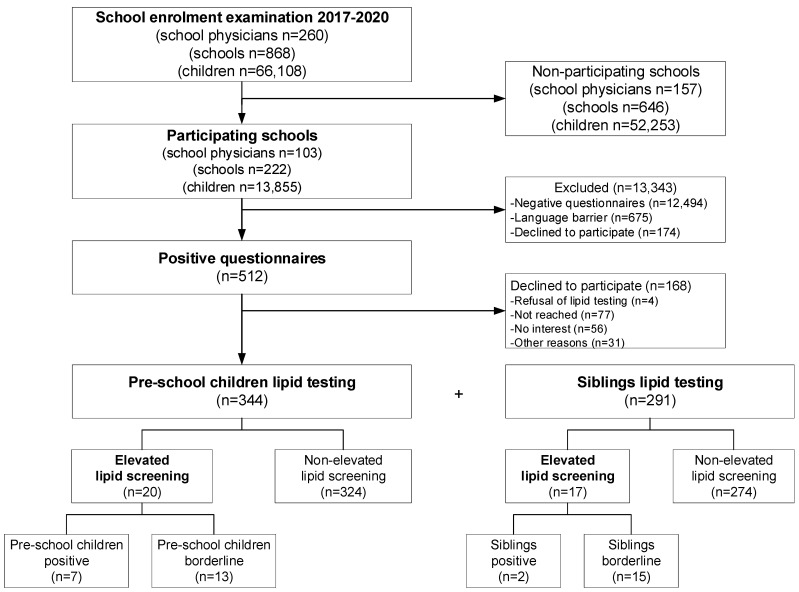
The flow diagram of screening strategy performed on pre-school children and siblings to detect FH.

Figure 1 shows the flow during the entire selective screening. 

In total, 103 (40%) school physicians and 222 (26%) elementary schools have participated in this selective FH-screening. Altogether, 13,855 (21%) families answered the school physician questionnaires and 512 (4%) families provided positive questionnaires. A questionnaire was defined as positive when one of the questions was answered with “yes” or one of the questions could not be answered or was unknown. Based on positive-answered questionnaires, a lipid screening test was performed on 344 (67%) pre-school children. Furthermore, a lipid screening test was conducted on 291 siblings (Table 1).

With the school physician questionnaire, the highest percentages in positive answered questions were detected regarding hypercholesterolemia (81%) and premature cardiovascular events (64%). Xanthomas or xanthelasma were present only in a low number of parents (4%). 

The parents’ questionnaire indicated that mothers’ median age was 36 years (range: 21–50 years) and had a median body mass index (BMI) of 24 kg/m^2^ (range: 17–53 kg/m^2^). Mothers were from 41 different countries of birth and the majority came from Austria (44%), followed by Turkey (9%) and Serbia (6%). The fathers’ median age was 40 years (range: 23–64 years) and had a median BMI of 27 kg/m^2^ (range: 19–49 kg/m^2^). Fathers were from 39 different countries of birth and most of the fathers came from Austria (40%), followed by Turkey (13%) and Serbia (8%).

More fathers (42%) than mothers (23%) were affected by a known hypercholesterolemia and adequate pharmacotherapy treatment was very low in both fathers (16%) and mothers (3%). With the same percentage of 2% in both parents, xanthomas and/or xanthelasma were present. More fathers (10%) than mothers (3%) suffered from a coronary heart disease. More first- and second-degree relatives of mothers (58%) than fathers (45%) had hypercholesterolemia. A premature coronary heart disease was determined in 30% of maternal and in 15% of paternal close relatives (Table 2). 

### 3.2. Pre-School Children and Their Siblings

Data were obtained from 344 (52% male) pre-school children with a median age of 6 years (range: 5–8 years) and from 291 (44% male) siblings with a median age of 7 years (range: 1–28 years) (Table 3).

The pre-school children had up to four siblings. In our study population, no xanthomas, xanthelasmata, arcus lipoides, or any cardiovascular comorbidities were present. Lipid screening test was performed on 635 children, of whom 344 (54%) were pre–school children and 291 (46%) were siblings. In tested pre-school children, 20 (6%) individuals had elevated lipids with a mean LDL-C of 155 ± 29 mg/dL (range: 111–216 mg/dL), non–-HDL-C of 180 ± 24 mg/dL (range: 150–325 mg/dL), and total cholesterol level of 242 ± 29 mg/dL (range: 206–313 mg/dL). Out of these index children, 7 (2%) children were detected to be positive with a mean LDL-C of 185 ± 24 mg/dL, non-HDL-C of 206 ± 19 mg/dL, and total cholesterol level of 273 ± 28 mg/dL. Borderline values were found in 13 (4%) pre-school children with a mean LDL-C of 139 ± 14 mg/dL, non-HDL-C of 166 ± 11 mg/dL, and total cholesterol level of 225 ± 11 mg/dL. Out of all 291 tested siblings, 17 children (6%) showed elevated lipids with a mean LDL-C of 144 ± 19 mg/dL, non-HDL-C of 174 ± 19 mg/dL, and a TC of 235 ± 24 mg/dL. Out of this group, 2 (1%) children were detected to be positive with a mean LDL-C of 183 ± 3 mg/dL, non-HDL-C of 207 ± 7 mg/dL, and total cholesterol level of 273 ± 13 mg/dL. 15 (5%) siblings were detected to be borderline with a mean LDL-C of 139 ± 12 mg/dL, non-HDL-C of 170 ± 15 mg/dL, and total cholesterol level of 230 ± 20 mg/dL. No gender differences have been detected in either group, in pre-school children and siblings (*p* > 0.05).

### 3.3. Venous Blood Measurement and Genetic Testing

If a pre-school child or one of their siblings had an elevated lipid screening test, the entire family was invited to the specialized lipometabolic disorder outpatient clinic. Thus, the number of children tested per family varied from one index child to several siblings. In the case of a positive family history, genetic testing for FH was offered to all children in the family to preclude a genetic modification even in siblings with cholesterol levels within the normal range. Therefore, venous lipid status and genetic testing were performed on 54 children (28 pre-school children and 26 siblings). No significant differences between capillary and venous blood lipid measurements were detected (*p* > 0.05) and could be seen as natural fluctuations (Table 4 and Table 5). 

Altogether, eight children (five pre-school children and three siblings) were tested positive for known FH causing genetic mutations. Four pre-school children and three siblings were heterozygous for LDL-receptor mutations; one pre-school child was for an *APOB* mutation (Table 4). 

Additionally, one pre-school child carried a known mutation on *LDLR* which was not clearly associated with familial hypercholesterolemia. In six children, new genetic variants on FH causing genes which were not previously described in the mutation database could be identified. Two pre-school children and two siblings showed heterozygous LDL-receptor variants and one pre-school child showed a heterozygous *PCSK9* variant. Moreover, one pre-school child with a known *PCSK9* mutation associated with low LDL-C levels and an additional unknown variant on the LDL-receptor gene showed definitely elevated LDL-C concentrations (Table 5). 

Three children were tested negative for FH, but showed heterozygous *LDLRAP1* gene variants. For *LDLRAP1*, a genetic alteration is autosomal recessive inherited. Therefore, the *LDLRAP1* gene in homozygous form would be related to FH (Table 6).

In the remaining tested children, no disease-associated variant in the *LDLR*, *PCSK9*, *APOB*, *APOE*, *STAP1*, or *LDLRAP1* gene was found.

## 4. Discussion

For pediatric populations, different screening strategies for familial hypercholesterolemia are available [1,12,14,15,16,17,18,19,20,21]. However, the tool that we have established to detect this inherited disease is unique. To reach such young children, the encouragement of health ministry and/or primary pediatricians is needed. In Austria, this support is not available; there is also no compulsory vaccination program to detect patients suffering from FH. Therefore, we have developed a feasible strategy with school physicians to identify young individuals with familial hypercholesterolemia in Vienna. This is the first screening program on familial hypercholesterolemia performed in Austrian children. 

### 4.1. Selective Screening Strategy

The markedly elevated LDL-cholesterol values are increased from birth, and it makes sense to start screening for familial hypercholesterolemia very early in life, particularly because children are the most underdiagnosed group [2,22]. We were able to show within our study that siblings were screened as positive and borderline at the age of one year.

Based on all participating screening schools, a majority of 90% provided negative-answered questionnaires and therefore represented no FH-risk. For lipid screening testing, pre-school children and their siblings were invited. All families who came to the lipid screening test accepted this offer and thus no siblings of the invited pre-school children were missing.

With the lipid screening test, the same percentage of 94% in pre-school children as well as in siblings received the pleasant information that they were not affected by familial hypercholesterolemia. In the pre-school children as well as in the siblings, the same percentage of 6% was detected to have an elevated lipid screening test. We know from literature that as soon as one index patient is identified, up to eight other family members suffer from FH and about nine family members are not affected by this disease [1,23]. Hence, our study sample has the potential to detect up to 160 family members affected by FH. In coherence with the literature [17], we are convinced that FH-screening performed on children is important for the detection of other relatives with this disease.

So far, the prevalence of familial hypercholesterolemia in Vienna is not estimated. However, based on the 13,855 families who completed the school physician questionnaire and calculating with a prevalence of 1:500, 28 children with FH should have been identified and with a prevalence of 1:200, as many as 69 children should have been detected. In fact, we identified 20 pre-school children and 17 siblings with positive lipid screening. Of these, in five pre-school children and three siblings known FH mutations could be found. There might be several reasons for the lower detection rate in our sample: (i) language barrier, (ii) refusal of study participation, (iii) no interest (iv) families were not reached (e.g., wrong contact details), (v) false negative questionnaires, and (vi) the fact that it is a selective screening model and not a universal screening approach.

With our screening, the awareness of this common inherited disease increases within a family and relatives are more likely to be tested. Knowledge about this autosomal dominant hereditary disorder and the experience within a family history for premature cardiovascular disease are factors which have an impact on the adherence to treatment and lifestyle recommendations [24]. If parents and/or other relatives have not yet been screened and tested for familial hypercholesterolemia and/or have not received adequate treatment, we have recommended them to visit lipid metabolic experts for adults. Therefore, we have created a list of experts for familial hypercholesterolemia in adults in Vienna and all other federal states in Austria and provided it to all families. 

With our study, we were able to show that parents who already suffered from a known hypercholesterolemia unfortunately did not receive adequate pharmacological treatment. This has adverse effects, as untreated men and women with familial hypercholesterolemia have a 30–50% risk of a cardiac event by the age of 50 (for women) or 60 years (for men) [25]. It must be considered that most parents of school children have not yet reached this age. Thus, lipid-lowering therapy is essential for the prevention of any cardiac event [26]. In particular, young patients with heterozygous familial hypercholesterolemia show no symptoms [1]. This is the main reason that the detection of this inherited disease in childhood is difficult. Our study population showed no clinical signs of familial hypercholesterolemia, which indicated that this selective FH-screening might be an appropriate prevention tool, especially in the early prevention of coronary heart diseases. Moreover, with an early FH detection, affected children can start with lifestyle intervention by adherence to a healthy fat-modified diet, regular physical activity, and smoking and alcohol avoidance. These interventions are often effective in lowering LDL-C levels in young heFH patients [3,8,27,28]. Furthermore, if necessary, lipid lowering medication can be started in childhood with a beneficial effect on the lifelong cumulative exposure to LDL-C [8,26].

Based on the lipid screening test, the identified children were invited for genetic testing and received regular visits at the outpatient clinic for lipometabolic disorders.

### 4.2. Benefits of This Type of Lipid Screening Test

The main benefit of the lipid screening test was that the phased plan with questionnaires and capillary puncture with immediate lab measurement could be performed easily. For lipid measurement capillary blood, only one to two drops from finger prick, was needed. To get proper results, it is important that the first blood drop was not used and was wiped away as it might be contaminated with tissue fluids leading to incorrect results. Moreover, it was essential to clean the finger prior to each puncture with alcohol and not to milk the finger, which might also result into erroneous blood findings.

The cut-off value of LDL-C with 160 mg/dL is based on the Simon Broome criteria cutoff value (4 mmol/L) for the diagnosis of FH in children aged 16 years or younger [25]. We defined lower values of ≥130–160 mg/dL as borderline positive according to Wiegman et al. [8]. FH is suspected if one parent has a genetic diagnosis and the child has an LDL-C ≥ 130 mg/dL [8]. The Joint British Societies’ guidelines (JBS3) [29] and the United Kingdom National Institute for Health and Clinical Excellence (NICE) [30] announced non-HDL-C for identification of dyslipidemia [31]. Based on the assumption that non-HDL-C is approximately 1.24 × LDL-C [29,32], we established our non-HDL-C cutoff threshold as ≥190 mg/dL for positive patients and ≥160 mg/dL for borderline individuals.

The main parameter LDL-cholesterol is dependent on triglyceride levels as it is calculated according to the Friedewald formula: LDL-C (mg/dL) = (TC) − (HDL-C) − (TG/5) [33]. In general, triglycerides should not exceed ≥ 400 mg/dL, otherwise LDL-C cannot be calculated using this equation [33]. Thus, the best option is to test individuals fasting. Another requirement was that other lipoproteins such as total cholesterol and HDL-C must be within the measuring range of the device. However, the analyzer comprises a large measuring range and individuals can be measured in a non-fasting state. The families were invited for a further visit if a laboratory value could not be measured. In coherence with our previous publication [13], we were able to show that the device measures valid results. No significant difference between cholesterol screening tests from capillary blood compared to venous blood measurement was observed. Although the differences were not significant, it is important to confirm the results from the finger prick test with a venous blood sampling.

### 4.3. Outcome of Genetic Testing

The diagnosis of familial hypercholesterolemia was genetically confirmed in eight children for known disease-causing mutations. One *LDLR* mutation, which was not clearly associated with FH, was detected in a child with elevated LDL-C. In six children, unknown variants on the FH causing LDL-receptor gene and *PCSK9* gene were detected. So far, the disease causality of these genetic changes is unclear, but may be associated to the LDL-C elevations. The described unknown heterozygous variants of the *LDLRAP1* gene could not be seen as disease-relevant because FH caused by *LDLRAP1* is an autosomal recessive inherited disease. All other individuals showed no genetic variants of genes related to familial hypercholesterolemia. 

Our results are in compliance with the literature. In general, the underlying genetic defects of familial hypercholesterolemia are caused by the *LDLR* gene in 90%, by the *APOB* gene in 5–10%, and the *PCSK9* gene in less than 1% of cases, and origination from the *LDLRAP1* gene is very rare [2,6]. It is particularly important to note that the prevalence and relative frequency of certain mutations vary considerably by geographic area and ethnic group (e.g., the relative frequency of *APOB* mutations ranges from 0% in Greece to 39% in the Czech Republic) [34]. In children, more than 90% of FH mutations have been already detected [35,36]. However, 10% have not been identified yet and approximately 5% to 30% of phenotypic FH cases might be from mutations in genes which are not identified yet or are based on a polygenic cause [7,36,37]. The following list includes some conditions or circumstances that might explain the remaining 5 to 30% of patients diagnosed with FH but without identifiable causative mutations: (i) the causative mutations might be located in regions that are not covered by the sequencing protocol, (ii) mutations within sequenced regions that are not yet known or understood (iii) epigenetic alterations that are not detected by conventional sequencing, (iv) misclassified secondary hypercholesterolemia caused by hidden underlying disorders (e.g., endocrinologic disorders, such as hypothyroidism), (v) phenotypic outliers without explanatory mutations, or (vi) polygenic FH [2,38]. Patients with FH without any mutation have a six-fold risk and those with a mutation have a 22-fold increased risk of CVD [39]. 

### 4.4. Limitations

There are over 200 public elementary schools in Vienna, for which around 60 school physicians are responsible every year. The missing resources of school physicians is a fundamental problem. School physicians have only approximately ten minutes to check a child during the pre-school examination. Even though the selective screening is based only on three questions, it is time-consuming for school physicians to screen families and to inform them as well as to provide them further information. The number of school physicians in Vienna is not consistent over the years and almost all school physicians are responsible for several schools and have an increased workload under time pressure. This was the main reason that 60% of school physicians were not able to support the screening, and, particularly during the study period, the decreasing number of schools willing to handle the FHkids package underlines this serious shortage. 

A very important factor is the motivation of school doctors and parents to actively participate in the screening. Before study initiation, all Viennese school doctors were trained. The school physicians received a presentation on the topics of familial hypercholesterolemia and implementation of the study and training on the study documents with focus on the questionnaires. The year after, all school physicians received retraining. In addition, the successful results of the first year were presented. In subsequent years, no training activities were conducted. This could be a reason why the rate of participating school doctors or schools has declined. The coronavirus pandemic might be an essential factor that the participation numbers dropped in the screening. COVID-19 had an impact on school enrolment examination as well as on parents not wanting to take their children to the hospital for screening during the pandemic.

However, we are convinced that a selective screening for FH with the support of school physicians at school enrolment examinations is the appropriate strategy to reach Viennese pre-school children at a certain time point. A higher participation rate could be achieved if the lipid screening were supported by the health ministry and the number of school physicians were increased. A main issue is the language barrier; even though we have translated all documents into four different languages, families could not be adequately informed by school physicians. Out of all positive questionnaires, in 33%, the lipid screening test was not performed as the families had no interest, could not be reached, or the contact details were incorrect. There is still potential in increasing the response rate of families and to improve this selective screening strategy. More data are required, especially to determine the prevalence of familial hypercholesterolemia in children in Vienna. The next step is to expand this screening strategy to other Austrian federal states. For this purpose, we will involve the responsible heads of school physicians as well as known experts and clinical centers which are specialized on pediatric lipometabolic diseases in Austria.

## 5. Conclusions

We are convinced that an early identification of familial hypercholesterolemia individuals is essential. Screening is the key for early diagnosis and treatment of FH. The main issue is that especially children with heterozygous familial hypercholesterolemia do not suffer from any symptoms, which makes detection of this monogenetic disorder more difficult. Therefore, we have implemented a pre-school screening strategy with the support of school physicians in Vienna. We could identify 20 pre-school children and 17 siblings with an elevated lipid screening test. Full implementation of FH-screening in the pre-school examination visits in Vienna would help to detect high-risk children.

## Figures and Tables

**Table 1:** Outcomes of the selective FH-screening strategy.

	All Years 2017–2020	1st Year 2017	2nd Year 2018	3rd Year 2019	4th Year 2020
Number of schools	868	215	214	251	188
Participating schools	222 (26%)	76 (35%)	100 (47%)	32 (13%)	14 (7%)
Number of school physicians	260	63	67	69	61
Participating school physicians	103 (40%)	37 (59%)	35 (52%)	19 (28%)	12 (20%)
Number of children started school	66.108	18.152	15.214	15.223	17.519
Participating children	13.855 (21%)	6325 (35%)	3842 (25%)	2468 (16%)	1220 (7%)
Positive questionnaires	512 (4%)	229 (4%)	228 (6%)	37 (1%)	18 (1%)
Pre-school children lipid testing	344 (67%)	133 (58%)	156 (68%)	37 (100%)	18 (100%)
Pre-school children elevated LDL-C/non-HDL-C	20 (6%)	9 (7%)	3 (2%)	6 (17%)	2 (11%)
Pre-school children positive	7 (2%)	3 (2%)	1 (1%)	3 (8%)	0 (0%)
Pre-school children borderline	13 (4%)	6 (5%)	2 (1%)	3 (8%)	2 (11%)
Siblings lipid testing	291	85	154	35	17
Siblings elevated LDL-C/non-HDL-C	17 (6%)	4 (5%)	3 (2%)	4 (11%)	6 (35%)
Siblings positive	2 (1%)	0 (0%)	0 (0%)	2 (6%)	0 (0%)
Siblings borderline	15 (5%)	4 (5%)	3 (2%)	2 (6%)	6 (35%)

Results are presented as numbers and percentages (%). LDL-C = Low Density Lipoprotein Cholesterol; non-HDL-C = non-HDL Cholesterol.

**Table 2:** Answers of parents’ questionnaire.

	Mothers (*n* = 344)			Fathers (*n* = 344)		
Questions	Yes	No	Not Measured/ Unknown	Yes	No	Not Measured/ Unknown
Hypercholesterolemia	79 (23%)	211 (61%)	54 (16%)	143 (42%)	145 (42%)	56 (16%)
Statin intake	11 (3%)	329 (96%)	4 (1%)	57 (16%)	267 (78%)	20 (6%)
Xanthomas/xanthelasma	6 (2%)	333 (97%)	5 (1%)	7 (2%)	316 (92%)	21 (6%)
Coronary heart disease	11 (3%)	329 (96%)	4 (1%)	33 (10%)	296 (86%)	15 (4%)
Relatives-hypercholesterolemia	201 (58%)	124 (36%)	19 (6%)	155 (45%)	142 (41%)	47 (14%)
Relatives-premature heart attack/stroke	102 (30%)	201 (58%)	41 (12%)	52 (15%)	122 (36%)	170 (49%)

Results are presented as numbers and percentages (%).

**Table 3:** Tested pre-school children and their siblings.

	Pre-School Children	Pre-School Children Elevated Lipid Screening	Siblings	Siblings Elevated Lipid Screening
N	344	20	291	17
Male N (%)	178 (52%)	8 (40%)	127 (44%)	7 (41%)
Age (years)	6 (5–8)	6 (5–8)	7 (1–28)	4 (1–16)
Height (cm)	118 (100–145)	117 (110–127)	127 (68–190)	110 (68–176)
Weight (kg)	22 (13–55)	21 (17–34)	26 (7–90)	28 (7–70)
BMI (kg/m²)	16 (11–31)	16 (13–22)	17 (9–35)	17 (11–30)
LDL-C (mg/dL)	76 (14–216)	151 (111–216)	71 (8–185)	141 (111–185)
non-HDL-C (mg/dL)	100 (47–235)	175 (150–325)	103 (49–212)	172 (141–212)
TC (mg/dL)	159 (99–313)	235 (206–313)	160 (99–282)	230 (177–282)
HDL-C (mg/dL)	58 (22–101)	58 (42–86)	56 (20–101)	57 (27–100)
TG (mg/dL)	114 (44–510)	109 (47–249)	125 (44–491)	145 (44–247)
C/HDL ratio	2.7 (1.6–6.0)	4.0 (2.9–5.5)	2.9 (1.6–8.0)	3.8 (2.6–8.0)

Data are presented as number of children (percent) or as median (range). A lipid screening test was performed by capillary blood sampling. BMI = body mass index. C/HDL ratio = cholesterol/high density lipoprotein ratio; HDL-C = high density lipoprotein cholesterol; LDL-C = low density lipoprotein cholesterol; TC = total cholesterol; TG = triglycerides; non-HDL-C = non-HDL cholesterol.

**Table 4:** Pre-school children and siblings with positive genetic testing for known FH-causing mutations.

	Lipid Screening Test						Venous Blood Sampling				Genetic Testing
ID	TC (mg/dL)	LDL-C (mg/dL)	HDL-C (mg/dL)	TG (mg/dL)	Non-HDL (mg/dL)	C/HDL Ratio	TC (mg/dL)	LDL-C (mg/dL)	HDL-C (mg/dL)	TG (mg/dL)	Positive for Familial Hypercholesterolemia
18_2017	275	210	50	76	225	5.5	253	189	50	70	*LDLR: E4: c.662A>G, p.Asp221Gly in heterozygous (het)-form*
45_2017	281	193	74	72	207	3.8	245	164	69	61	*APOB: E26: c.10580G>A, p.Arg3527Gln in het-form*
87_2017	226	152	58	82	168	3.9	254	176	66	59	*LDLR: E6: c.858C>A, p.Ser286Arg in het-form*
92S3_2017	238	148	66	118	172	3.6	235	166	58	57	*LDLR: E5: c.798T>GA, p.Asp266Glu in het-form*
21_2018	260	161	79	99	181	3.3	301	209	81	54	*LDLR: E17:c.2483A>G, p.Tyr828Cys in het-form*
61S2_2018	194	123	50	105	144	3.9	179	118	45	82	*LDLR: E10: c.1414G>A, p.Asp472Asn in het-form*
19_2019	313	216	78	96	235	4	352	261	73	91	*LDLR: E10: c.1474G>A, p.Asp492Asn in het-form*
19S1_2019	264	181	52	157	212	5.1	262	196	49	87	*LDLR: E10: c.1474G>A, p.Asp492Asn in het-form*

C/HDL ratio = cholesterol/high density lipoprotein ratio; HDL-C = high density; lipoprotein cholesterol; LDL-C = low density lipoprotein cholesterol; TC = total cholesterol; TG = triglycerides; non-HDL-C = non-HDL cholesterol.

**Table 5:** Pre-school children and siblings with novel genetic variants on known FH causing genes.

	Lipid Screening Test						Venous Blood Sampling				Genetic Testing
ID	TC (mg/dL)	LDL-C (mg/dL)	HDL-C (mg/dL)	TG (mg/dL)	Non-HDL (mg/dL)	C/HDL Ratio	TC (mg/dL)	LDL-C (mg/dL)	HDL-C (mg/dL)	TG (mg/dL)	New Genetic Variants
96_2017	233	177	42	69	191	5.5	270	213	49	39	*LDLR: E11: c.1683-1690delGTGGCCCAinsCCCTATGTTCGCAGGACAGCCT, p.Gln561_Asn564delinsHisProArgTyrValArgArgThrAlaTyr in het-form; known mutation associated with low LDL-C: PCSK9: E1: c.137G>T, p.Arg46Leu in het-form*
105_2017	230	141	80	47	150	2.9	207	131	68	38	*PCSK9: E1: c.75C>T, p.Pro25= in het-form*
108_2017	218	129	54	176	164	4	219	151	42	131	*LDLR: E7: c.993C>T, p.Asp331 in het-form*
5S2_2017	230	157	44	143	186	5.2	235	159	51	127	*LDLR: I5: c.817+6C>T in het-form*
9_2019	297	187	86	120	211	3.5	277	196	61	100	*LDLR: E4: c.386A>C, p.Asp129Ala in het-form*
9S1_2019	282	185	80	84	202	3.5	286	201	71	70	*LDLR: E4: c.386A>C, p.Asp129Ala in het-form*
											**Mutation—not clearly associated with FH**
105_2018	241	155	57	147	184	4.2	265	203	47	76	*LDLR: I11: c.1706-10G>A in het-form*

C/HDL ratio = cholesterol/high density lipoprotein ratio; HDL-C = high density; lipoprotein cholesterol; LDL-C = low density lipoprotein cholesterol; TC = total cholesterol; TG = triglycerides; non-HDL-C = non-HDL cholesterol.

**Table 6:** Children with heterozygous *LDLRAP1* gene variants.

	Lipid Screening Test						Venous Blood Sampling				Genetic Testing
ID	TC (mg/dL)	LDL-C (mg/dL)	HDL-C (mg/dL)	TG (mg/dL)	Non-HDL (mg/dL)	C/HDL Ratio	TC (mg/dL)	LDL-C (mg/dL)	HDL-C (mg/dL)	TG (mg/dL)	New Genetic Variants
4S1_2019	203	128	60	74	143	3.4	202	137	52	65	*LDLRAP1: E7: c.742G>A, p.Val248Ileu in in het-form*
143_2018	217	121	71	127	146	3.1	222	145	62	74	*LDLRAP1: I1: c.88+31C>A in het-form*
143S1_2018	213	123	70	101	143	3.0	220	146	61	65	*LDLRAP1 I1: c.88+31C>A in in het-form*

C/HDL ratio = cholesterol/high density lipoprotein ratio; HDL-C = high density; lipoprotein cholesterol; LDL-C = low density lipoprotein cholesterol; TC = total cholesterol; TG = triglycerides; non-HDL-C = non-HDL cholesterol.

## Data Availability

The data presented in this study are available upon request from the corresponding author.
